# *In vitro* regeneration of *Portulaca grandiflora* Hook. and analysis of betalain content within *in vivo* plants

**DOI:** 10.5114/bta/209760

**Published:** 2025-09-22

**Authors:** Archana Srivastava, Aruna Joshi

**Affiliations:** 1 Department of Botany, Bhavan’s Sheth R.A. College of Science, Gujarat University, Ahmedabad 380001, Gujarat, India; 2 Department of Botany, Faculty of Science, The Maharaja Sayajirao University of Baroda, Vadodara 390002, Gujarat, India

**Keywords:** anatomy, betalain, callus, *in vitro* shoots, leaf explants, *Portulaca grandiflora*

## Abstract

**Background:**

Synthetic colors are widely used in the food and cosmetic industries to make products more appealing to consumers. However, the health hazards associated with synthetic colors have prompted their replacement with natural colors. *Portulaca grandiflora* is a promising candidate for natural color extraction, as it is rich in betalains. This study presents a reliable, reproducible three-step regeneration protocol for this plant and analyzes its betalain content.

**Materials and methods:**

*In vitro* shoot cultures were established on Murashige and Skoog (MS) medium supplemented with different plant growth regulators. Anatomical studies were conducted to determine the stages of shoot primordium development. Betalains were extracted from *in vivo* plants using 60% methanol and subjected to spectrophotometric analysis. The effect of sodium ascorbate on betalain stability was also evaluated.

**Results:**

Juvenile leaf explants regenerated shoots on MS medium supplemented with 10 μM 6-benzyladenine and 5 μM indole-3-acetic acid. Shoots were multiplied with 20 μM BA (6.25 ± 0.85 shoots/explant), and elongation was achieved with 5 μM gibberellic acid (GA_3_) (8.2 ± 0.37 shoots/explant). Shoot primordia developed from well-organized meristemoid cells. The betalain content in the stem was 26.66 ± 0.19 mg/100 g, but this pigment degraded within 24 h (42.19% degradation). The addition of 50 mM sodium ascorbate prevented betalain degradation, even after 24 h.

**Conclusion:**

This study reports a regeneration protocol from juvenile leaf explants and demonstrates that betalain stability in the stem can be maintained with 50 mM sodium ascorbate.

## Introduction

The genus *Portulaca* (family *Portulacaceae*) comprises over 100 species, distributed across tropical and subtropical regions (Gilbert and Phillips 2000). *Portulaca grandiflora*, commonly known as moss rose or office hour plant, is widely cultivated in gardens and landscapes as an ornamental plant. Its flowers are large and showy, appearing in several shades (red, pink, magenta, yellow, orange, white, etc.), and may occur in single, semidouble, or double forms, blooming during summer. The plant has a prostrate growth habit, and its stem is red due to the presence of betalain pigment.

This species is also a source of various phytochemicals, including phenols, sterols, flavonoids, and polysaccharides (Anghel et al. 2013). The betalain pigment has multiple medicinal benefits for human health, such as antidiabetic, anti-inflammatory, antioxidant, lipid-lowering, and antiobesity effects (Calvi et al. 2022). Nutritionists and cosmetologists are interested in this pigment because it acts as a natural colorant. Synthetic colors, associated with several health hazards, are increasingly being replaced with natural alternatives to enhance the appeal of products. Betalains are considered safe, water-soluble, and require no chemical modification before use in food (Polturak and Aharoni 2018).

Beetroot is a widely used source of betalains; however, it presents certain disadvantages. Beetroot betalains contain geosmins and pyrazines, which are aromatic terpene derivatives (Wang et al. 2020a). These compounds impart an unpleasant earthy and musty odor to food products, negatively affecting consumer satisfaction (Wang et al. 2020b). Moreover, beetroot may carry microorganisms from the soil that can compromise product quality. Therefore, alternative sources of betalains need to be explored, and *P. grandiflora* is a promising candidate.

Plant tissue culture is a method by which the primary and secondary metabolite content of plants can be enhanced. In *Portulaca*, different metabolites have been increased *in vitro* using several techniques, such as elicitation, mutagen treatment, and hairy root cultures (Azni et al. 2021; Srivastava and Joshi 2021; Moghadam et al. 2014). Reproducible regeneration protocols are required to harness these important metabolites through such manipulations.

**Figure 1:**
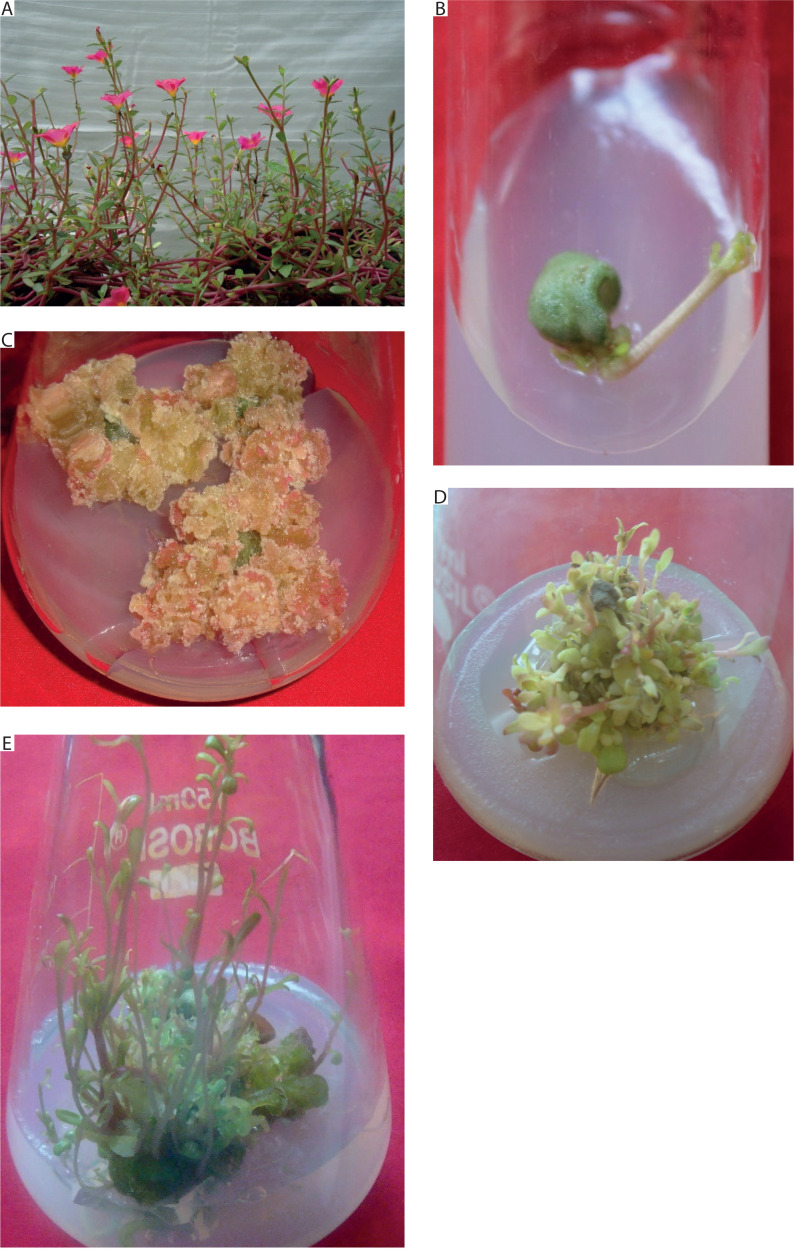
Response of leaf explant of *Portulaca grandiflora* inoculated on MS medium supplemented with different plant growth regulators. **A)** Experimental plant. **B)** *In vitro* shoot induction from mature leaf explant at 10 μM 6-benzyl adenine (BA) and 5 μM indole-3-acetic acid (IAA) after 4 weeks. **C)** Induction of green-red callus from mature leaf explant on medium supplemented with 10 μM BA and 10 μM IAA after 8 weeks. **D)** Shoot multiplication on medium supplemented with 20 μM BA after 2 weeks of transfer from juvenile leaves. **E)** Elongation of *in vitro* shoots in medium supplemented with 5 μM gibberellic acid (GA_3_) after 2 weeks

*In vitro* regeneration has been reported from *P. oleracea* and *P. pilosa* (Safdari and Kazemitabar 2009; Sedaghati et al. 2019; Chen et al. 2020; Srivastava and Joshi 2021). In *P. grandiflora*, regeneration from hypocotyl explants was reported by Rossi-Hassani and Zryd (1995) and Bhuiyan and Adachi (2002). Cruz et al. (2019), Safdari and Kazemitabar (2010), and Srivastava and Joshi (2009) regenerated *in vitro* shoots from nodal explants. Furthermore, Safdari and Kazemitabar (2010) successfully induced shoots from callus derived from the lanceolate leaf form of *P. grandiflora*. To the best of our knowledge, a regeneration protocol for *P. grandiflora* plants with obovate succulent leaves and magenta flowers (Figure 1A) has not yet been reported.

The aim of the present study was to develop a reproducible regeneration protocol for *in vitro* shoot development from leaf explants of *P. grandiflora* (plants with obovate succulent leaves and magenta flowers). The protocol described here can be used to enhance the biochemical synthesis of betalains within *in vitro* shoots. We report a three-step protocol for *in vitro* regeneration of *P. grandiflora* (plants with magenta flowers and obovate succulent leaves) from leaf explants, along with an analysis of the anatomical stages leading to shoot development. The betalain content in gardengrown plants was evaluated, and the potential of betalain production from *in vitro* shoots was explored.

## Materials and methods

### Source of explants

Seeds of *P. grandiflora* were obtained from the local market of Vadodara, Gujarat, India. Twenty pots filled with garden soil were maintained in the botanical garden of The Maharaja Sayajirao University of Baroda, Vadodara, Gujarat, India. A lot of seeds, each containing 10 seeds, were shallowly mixed with soil per pot and kept in bright sunlight. The seeds were sown in February 2016 and watered regularly without the application of pesticides or fertilizers. Fully developed plants were available by the middle of May, which were used for different experimental analyses. The plants were multiplied by stem cuttings from the parent experimental plants. As the stem of *P. grandiflora* is herbaceous, 3–4 cm segments were cut with a sharp, sterilized blade. The bottom 3–4 leaves were removed, and these stem cuttings were planted in fresh pots. Within 4–5 weeks, the cuttings developed into healthy plants. Multiplication through stem cuttings was carried out regularly throughout the experimental period to ensure sufficient fresh biomass for betalain analysis.

The leaves of *P. grandiflora* are sessile, glabrous, obovate, and alternately arranged. The young leaf at the apex of the stem was designated as the first nodal leaf, or juvenile leaf, while leaves present at or beyond the 5^th^ node were considered mature leaves. Tissue culture experiments were performed using juvenile leaves as well as leaves from the 5^th^ or 6^th^ node. The response of juvenile leaf explants to plant growth regulator (PGR) concentrations that induced maximum shoot production in mature leaf explants was also evaluated. Thus, a comparison of the responses of juvenile (1^st^ node) and mature (5^th^ node) leaf explants under the same medium composition (concentration of PGRs that induced maximum *in vitro* shoots) was conducted.

The source of leaf explants for tissue culture experiments was these plants potted in the botanical garden of The Maharaja Sayajirao University of Baroda, Vadodara, Gujarat, India. Leaves were washed with a mild detergent and rinsed under running water for one hour to remove soil or dust particles. They were then surfacesterilized with 0.1% HgCl_2_ for 3 min, followed by several washes with sterile distilled water. A 1 cm^2^ portion of each explant, cut from the center of the leaf (including the midrib) with a sterile blade, was inoculated onto the prepared medium under a laminar airflow cabinet.

### Preparation of medium

Tissue culture studies were initiated using Murashige and Skoog (MS) medium (Murashige and Skoog, 1962), supplemented with 3% sucrose, 0.8% agar, and different PGRs. Chemicals, sucrose, and agar required for the preparation of MS medium and other experimental analyses were purchased from Sisco Research Laboratories (SRL, Mumbai, Maharashtra, India).

The following PGR combinations were used for *in vitro* shoot regeneration, each tested at four concentrations (0.5, 2.5, 5.0, and 10.0 μM):

- 6-benzyladenine (BA) + kinetin (Kin),
- BA + indole-3-acetic acid (IAA),
- BA + 1-naphthaleneacetic acid (NAA),
- BA + 2,4-dichlorophenoxy acetic acid (2,4D),
- Kin + IAA,
- Kin + NAA,
- Kin + 2,4D.

All PGRs used in this study were purchased from Hi-Media Laboratories Pvt. Ltd., Mumbai, Maharashtra, India.

Shoot multiplication was carried out on basal MS medium supplemented with BA at 5, 10, 15, 20, 25, and 30 μM. Shoot elongation was performed on basal MS medium supplemented with gibberellic acid (GA_3_) at 0.5, 2.5, 5.0, and 10 μM. After the addition of PGRs, the pH of the medium was adjusted to 5.8. Agar (0.8%) was used as the gelling agent. The medium was warmed on a hot plate (Remi Elektrotechnik Ltd., Mumbai, Maharashtra, India), dispensed into culture tubes or 150 ml Erlenmeyer flasks, and sealed with autoclavable caps. Sterilization was carried out by autoclaving at 121°C for 20 min at 15 lb/square inch.

Since GA_3_ is thermolabile, it was freshly prepared in sterile distilled water, passed through a filter sterilization assembly with a pore size of 0.2 μm, and added at the required concentration to the molten autoclaved medium. The entire procedure was performed under the sterile environment of the laminar airflow cabinet.

The inoculated explants were maintained on different medium combinations in culture rooms fitted with cool white fluorescent lamps (2000 lux in each 1 m^2^ culture rack) with a 12-h photoperiod at 25°C. Lamps for the culture racks were procured from Signify Innovations India Ltd., Mumbai, Maharashtra, India.

### Anatomy studies of the developing in vitro shoots

For anatomical studies, regeneration was induced from juvenile leaves inoculated on MS medium supplemented with 10 μM BA and 5 μM IAA. The anatomical regeneration pattern was studied at 7-day intervals. Developing explants were harvested weekly and fixed in formaldehyde-acetic acid-alcohol (FAA). The FAA fixative consisted of 40% formaldehyde, acetic acid, ethanol, and distilled water in the ratio of 10 ml : 5 ml : 50 ml : 35 ml, respectively (Moreno-Sanz et al. 2020).

Histological slides were prepared from transverse sections of first-node leaves of *in vivo* plants and 2–3 developing explants per week (inoculated on 10 μM BA and 5 μM IAA medium composition), following the method of Johansen (1940). FAA was removed from the samples by washing them in an ascending tertiary butyl alcohol (TBA) series (30%, 50%, 70%, 90%, and 100%) purchased from Baroda Chemical Industries Limited (BCIL), Vadodara, Gujarat, India. Each step of the TBA series treatment was carried out for 30–35 min at 20–25°C.

The dehydrated tissues were then cleared in xylene to remove TBA. Samples were placed in a 100 ml coupling jar filled with xylene for 10 min at room temperature (20–25°C), and the procedure was repeated twice for complete clearing. The cleared tissues were infiltrated with molten paraffin wax (melting point 56–58°C) and embedded in paraffin blocks. Sections of 15 μm thickness were cut from the embedded tissues using a rotary microtome (Leica Biosystems, Nussloch, Germany).

The sections were mounted on glass slides, dewaxed, and stained with hematoxylin and eosin (SRL, India). They were then mounted in DPX (Distreneplasticizer-xylene), and photomicrographs were captured with a Leica Research Microscope (Leica Microsystems, a Division of Danaher, Wetzlar, Germany).

### Extraction of betalain

*In vivo* plants (approximately 1–1.5 kg fresh weight) were harvested from pots maintained in the botanical garden of the University. These plants were separated into two groups: the first consisting of whole vegetative plants (roots + leaves + stems), and the second consisting of stems only (with roots and leaves removed). Samples were washed under running tap water for 1 h and air-dried overnight. They were then transferred to a hot-air oven (Thermolyne Corporations, Iowa, USA) at 60°C for 2 days.

Dried samples (10 g) were ground into a fine powder using a mortar and pestle, and betalains were extracted with 100 ml of 60% methanol (BCIL, India) in a porcelain Buchner funnel (Cole-Parmer India, Mumbai, Maharashtra, India) for 30 minutes at room temperature (Kugler et al. 2004). The extract was subjected to spectrophotometric analysis (λ = 400–600 nm) using a UVVisible Spectrophotometer (PerkinElmer, Connecticut, USA) equipped with Lambda 25 software.

The extract was diluted with McIlvaine’s buffer [pH 6.0 (McIlvaine 1921)] to obtain an absorption value of 0.9 ≤ *A* ≤ 1.0 at a wavelength scan of 400–600 nm. The amount of betalains was calculated according to El-Ashry et al. (2020) using the following equation:

Betalain content mg/g=A×(DF)×(MW)×Vd/(∈×L×W)

where *A* – absorption value at the absorption maxima, *Vd* – volume of extract sample (ml), *W* – weight of sample (g), *DF* – dilution factor, *L* – path length (1 cm) of the cuvette, *MW* – molecular weight, and ε – molar extinction coefficient.

The values for betacyanin are MW = 550 g/mol, ∈ = 60000 L/mol · cm in H_2_O, λ (wavelength) = 540 nm. The values for betaxanthin are MW = 340 g/mol, ∈ = 48000 L/mol · cm in H_2_O, λ = 480 nm. The content of betalains obtained as mg/g was converted to mg/100 g.

The betalain contents of both the whole plant and the stem were evaluated. Initial spectrophotometric analysis revealed that both samples (whole plant and stem) lost betalain pigment after 12 h of storage at 4°C. To improve stability, a stock solution of sodium ascorbate (1 M) was prepared in distilled water and added to the extracts at a final concentration of 50 mM (Kugler et al. 2004). After 24 h, the betalain content of stem extracts, with and without 50 mM sodium ascorbate, was analyzed using a UV-Visible Spectrophotometer (PerkinElmer, Connecticut, USA) equipped with Lambda 25 software. The difference in betalain content between the two treatments was compared to assess the role of sodium ascorbate in preventing pigment degradation and enhancing pigment stability.

Shoot cultures (explants containing *in vitro* shoots and shoot buds) obtained from plant tissue culture experiments were subcultured on multiplication medium. This medium consisted of MS medium supplemented with BA at concentrations of 5, 10, 15, 20, 25, and 30 μM. Shoot cultures from one flask were transferred into two to three fresh flasks containing multiplication medium (MS + 5–30 μM BA) every 4 weeks for a period of three months to increase biomass production.

Thereafter, the shoot cultures were collected with sterilized forceps, dried on a paper towel, wrapped in coarse filter paper, and kept in an oven at 60°C for 48 h. From this material, 10 g of dried *in vitro* shoot cultures (without any *in vivo* plant part) was used for betalain assessment, following the same procedure as described for whole plants and stems.

### Statistical analysis

Plant tissue culture experiments were performed with ten explants for each combination, and each experiment was repeated twice. The number of *in vitro* shoots was expressed as the mean ± standard error (SE). Statistically significant differences in the data (mean ± SE) for shoot multiplication and elongation were analyzed using one-way ANOVA, followed by posthoc Tukey’s HSD test (*p* ≤ 0.05).

The analysis of betalain content (whole plant and stem) was performed in triplicate. Betalain content after spectrophotometric analysis was expressed as the mean of triplicate values for both whole plant and stem in mg/100 g ± SE. The significant difference between betalain content of whole plants and stems treated with or without sodium ascorbate was evaluated using Student’s *t*-test at *p* = 0.01. Heat map matrices representing the percentage of leaf explants forming callus and shoot buds were generated using Google Colab (https://colab.research.google.com/).

## Results

### Regeneration of the in vitro plant

The experimental plants exhibited prostrate, decumbent stems, obovate succulent leaves, and singleform magenta flowers (Figure 1A). Regeneration from *in vivo* mature leaf explants was tested on 112 different medium compositions of PGRs, but well-developed shoots were achieved only on MS medium supplemented with 10 μM BA and 5 μM IAA.

Leaf explants inoculated on MS medium containing 0.5 μM BA and IAA (5–10 μM) produced little to moderate callus without shoot bud induction. Medium supplemented with 2.5 μM BA and IAA (2.5–10 μM) induced little to moderate white callus, along with shoot buds in 40–70% of explants. Increasing BA to 5 μM with IAA (0.5–10 μM) resulted in minimal callus formation; however, the proportion of explants forming shoot buds increased to 100% at this concentration. A similar morphogenic response (callus and shoot bud induction) was observed in explants inoculated on MS medium supplemented with 10 μM BA and 0.5–10 μM IAA.

At low concentrations of BA (2.5–5 μM) with IAA (2.5–10 μM), clusters of shoot buds were initiated by the 6^th^ week, with a response rate of 40–100%. A much faster response was recorded at 10 μM BA and 2.5–5 μM IAA, where 50–100% of explants formed shoot buds by the 3^rd^ week. Shoot buds were green, shiny, and emerged from the abaxial surfaces of explants. These buds proliferated into *in vitro* shoots (2.31 ± 0.86) by the end of the 4^th^ week on medium supplemented with 10 μM BA and 5 μM IAA (Figure 1B). A greenish-red callus developed from explants cultured on medium supplemented with 10 μM BA and 10 μM IAA (Figure 1C and Table 1).

**Table 1:** *In vitro* shoots (mean ± SE), % share of explants forming shoot buds, and % share of explants inducing callus from mature leaf explant of *Portulaca grandiflora* on MS medium supplemented with 6-benzyl adenine (BA) and indole-3-acetic acid IAA (μM) after 4 weeks

PGR’s [μM]		Callus	% Explants forming shoot buds	Number of shoots/explants (mean ± SE)
BA	IAA			
0.5	0.5	–	–	–
0.5	2.5	+	–	–
0.5	5	+	–	–
0.5	10	++	–	–
2.5	0.5	–	–	–
2.5	2.5	+	40	–
2.5	5	++	50	–
2.5	10	++	70	–
5	0.5	+	30	–
5	2.5	+	60	–
5	5	+	100	–
5	10	+	100	–
10	0.5	+	40	–
10	2.5	+	50	–
10	5	++	100	2.31 ± 0.86
10	10	++	100	–

– = No response, + = little callus, ++ = moderate callus

MS medium – Murashige and Skoogs medium, 1962

Since explant age is a crucial factor for regeneration, mature leaf explants were replaced with juvenile leaves. Juvenile leaves obtained from the first node of plants were inoculated on MS medium containing 10 μM BA and 5 μM IAA, the concentration selected because mature leaves produced *in vitro* shoots only under this concentration. Shoot buds, a cluster of dense short shoots, and 2.7 ± 0.15 well-developed shoots were induced by the juvenile leaf explant by the end of the 6^th^ week on this combination of PGR. Thus, the mature leaves induced 2.31 ± 0.86 shoots only, while the juvenile leaves induced shoot buds, a cluster of dense short shoots, and 2.7 ± 0.15 well-developed shoots. This result depicted that the juvenile leaf gave a better response than the mature leaf in the induction of *in vitro* shoots.

### Multiplication and elongation of in vitro shoots

**Figure 2:**
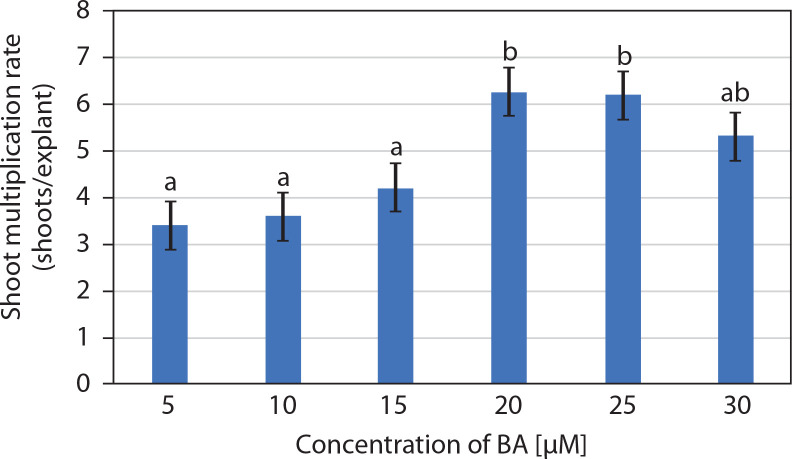
Effect of 6-benzyl adenine (BA) (μM) on the multiplication of *in vitro* shoots (mean ± SE) of *Portulaca grandiflora* after 2 weeks. Means followed by the same letters are statistically not significant with Tukey’s HSD test, *p* ≤ 0.05

The entire juvenile explant that induced 2.7 ± 0.15 well-developed *in vitro* shoots, clusters of short shoots, and shoot buds (on 10 μM BA and 5 μM IAA) was transferred to multiplication medium consisting of MS medium supplemented with BA (5–30 μM). Multiple shoots were formed from the adventitious shoot buds, and nodular green callus was occasionally observed between the newly developed shoots. The number of shoots per explant increased in MS medium supplemented with 5 and 10 μM BA, while medium containing 20 and 25 μM BA produced a similar number of shoots. Notably, 20 μM BA resulted in 6.25 ± 0.85 shoots per explant along with clusters of incipient shoots after 2 weeks. However, at 30 μM BA, a decline in the number of shoots was observed (Figures 1D and 2). Therefore, MS medium supplemented with 20 μM BA was selected as the optimum concentration for shoot multiplication.

**Figure 3:**
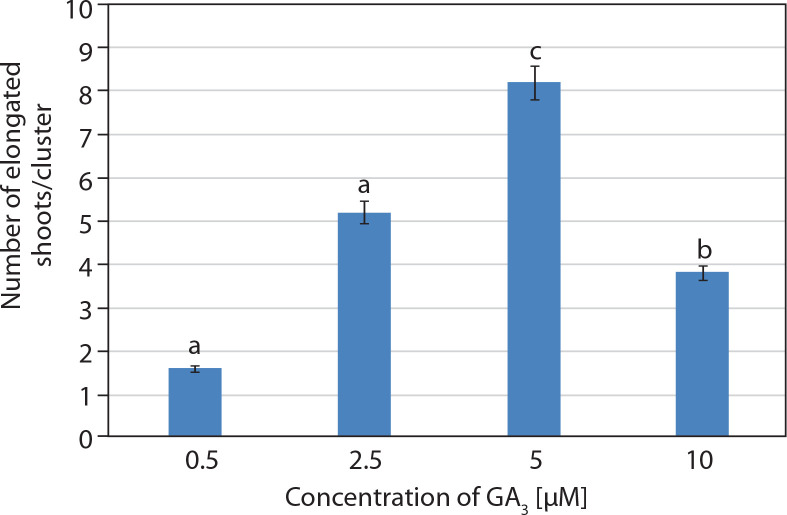
Effect of basal MS medium supplemented with gibberellic acid (GA_3_) (μM) on elongation of *in vitro* shoots/cluster (mean ± SE) in *Portulaca grandiflora* after 2 weeks. Means followed by the same letters are statistically not significant with Tukey’s HSD test, *p* ≤ 0.05

Shoot cultures grown on multiplication medium (MS + 20 μM BA) consisted of explants bearing several incipient or newly developed shoots. These explants were subsequently transferred to elongation medium containing basal MS medium supplemented with GA_3_ at different concentrations (0.5–10 μM). Visual observations revealed rapid elongation of incipient shoots within 1 week of inoculation. The best response was achieved at 5 μM GA_3_, with 8.2 ± 0.37 shoots/cluster after 2 weeks. Initially, the shoots appeared green, and by the 2^nd^ week, they had elongated and developed a pale pink coloration (Figures 1E and 3).

### Induction of callus from mature leaf explants

**Figure 4:**
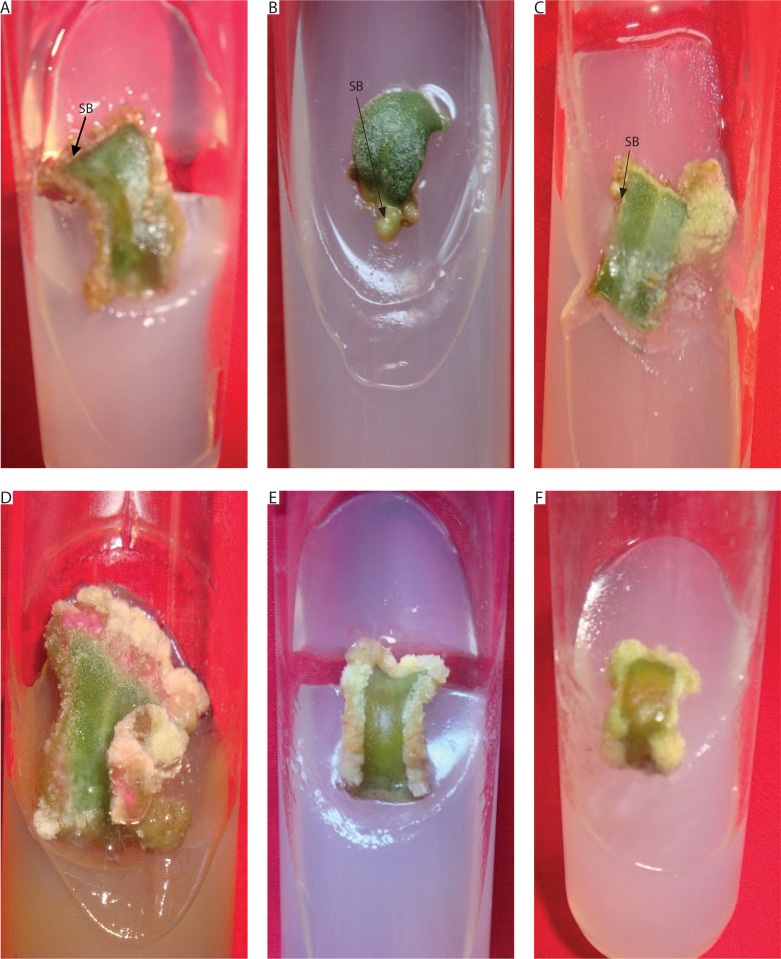
Induction of callus and shoot buds on mature leaf explants of *Portulaca grandiflora* inoculated on MS medium supplemented with different plant growth regulators. **A**) Shoot bud induction and callus initiation from mature leaf explant at 5 μM kinetin (Kin) and 2.5 μM indole-3-acetic acid (IAA) after 4 weeks. **B**) Shoot bud induction from mature leaf explant at 10 μM 6-benzyl adenine (BA) and 5 μM Kin after 4 weeks. **C**) Shoot bud and callus induction from mature leaf explant at 0.5 μM BA and 2.5 μM 1-naphthalene acetic acid (NAA) after 4 weeks. **D**) White and red mixed callus induction from mature leaf explant at 5 μM Kin and 2.5 μM NAA after 4 weeks. **E**) White friable callus induction from mature leaf explant at 5 μM BA and 10 μM 2,4-dichlorophenoxyacetic acid (2,4D) after 4 weeks. **F**) Little white green mixed friable callus induction from mature leaf explant at 5 μM Kin and 10 μM 2,4D after 4 weeks. SB – shoot buds

**Figure 5:**
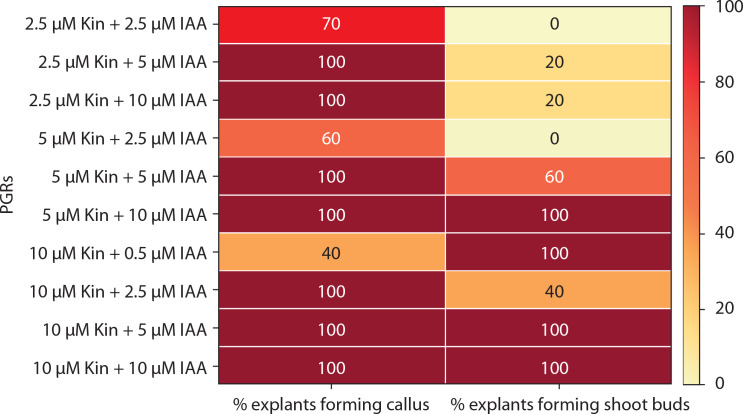
Heat map matrix displaying the combined effect of kinetin (Kin) and indole-3-acetic acid (IAA)-supplemented MS medium on the % share of explants forming callus and % share of explants forming shoot buds PGRs – plant growth regulators

The response of mature leaf explants of *P. grandiflora* was evaluated on MS medium supplemented with combined concentrations of other PGRs (Kin + IAA, BA + NAA, Kin + NAA, BA + 2,4D, Kin + 2,4D). Explants inoculated on medium with Kin and IAA produced white friable callus and shoot buds from the abaxial surfaces during the 4^th^ week of culture. At low concentrations of Kin (0.5 μM) and IAA (0.5–2.5 μM), negligible callus was formed. Kin at 2.5 μM with IAA (2.5–10 μM) induced little callus, but at 5–10 μM IAA, all explants (100%) produced callus, and 20% formed shoot buds. At higher concentrations of Kin (5–10 μM) with IAA (5–10 μM), 100% of explants formed callus, while 40–100% induced shoot buds (Figure 4A). However, these shoot buds failed to develop into *in vitro* shoots (Figure 5). Medium supplemented with 0.5 μM BA and 0.5 μM Kin induced roots (1.82 ± 0.23) in 80% of explants. At higher cytokinin concentrations (5–10 μM BA with 0.5–10 μM Kin), clusters of shoot buds were observed in 20–50% of cultures by the 3^rd^ week. Again, these shoot buds failed to regenerate into shoots (Figure 4B; Supplementary Table 1).

**Figure 6:**
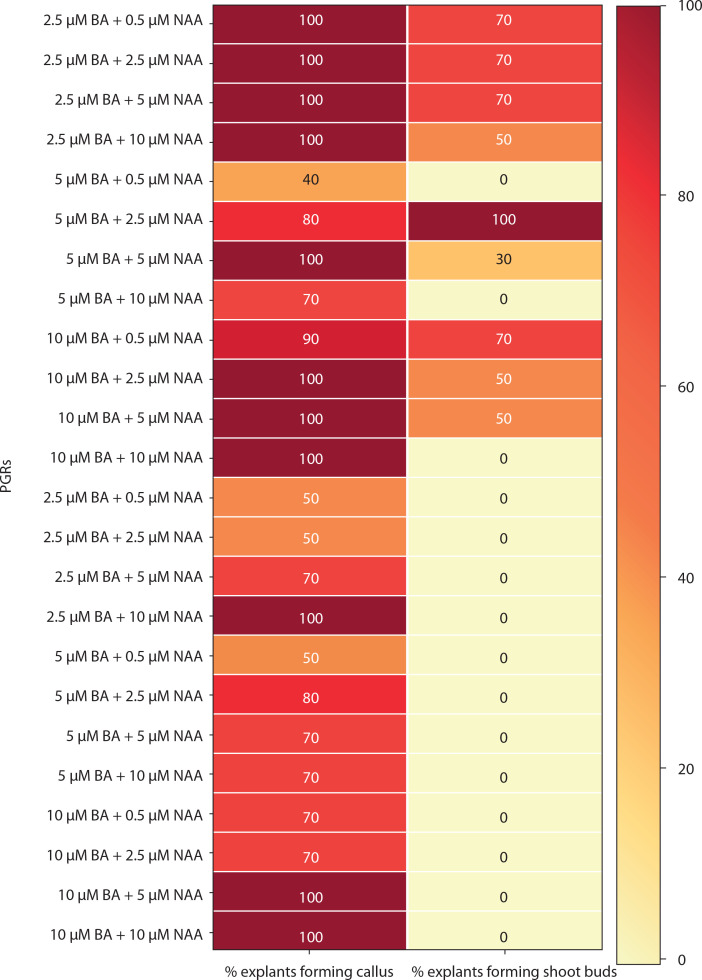
Heat map matrix displaying the combined effect of 6-benzyl adenine (BA)/kinetin (Kin) and 1-naphthalene acetic acid (NAA)-supplemented MS medium on the % share of explants forming callus and % share of explants forming shoot buds PGRs – plant growth regulators

**Figure 7:**
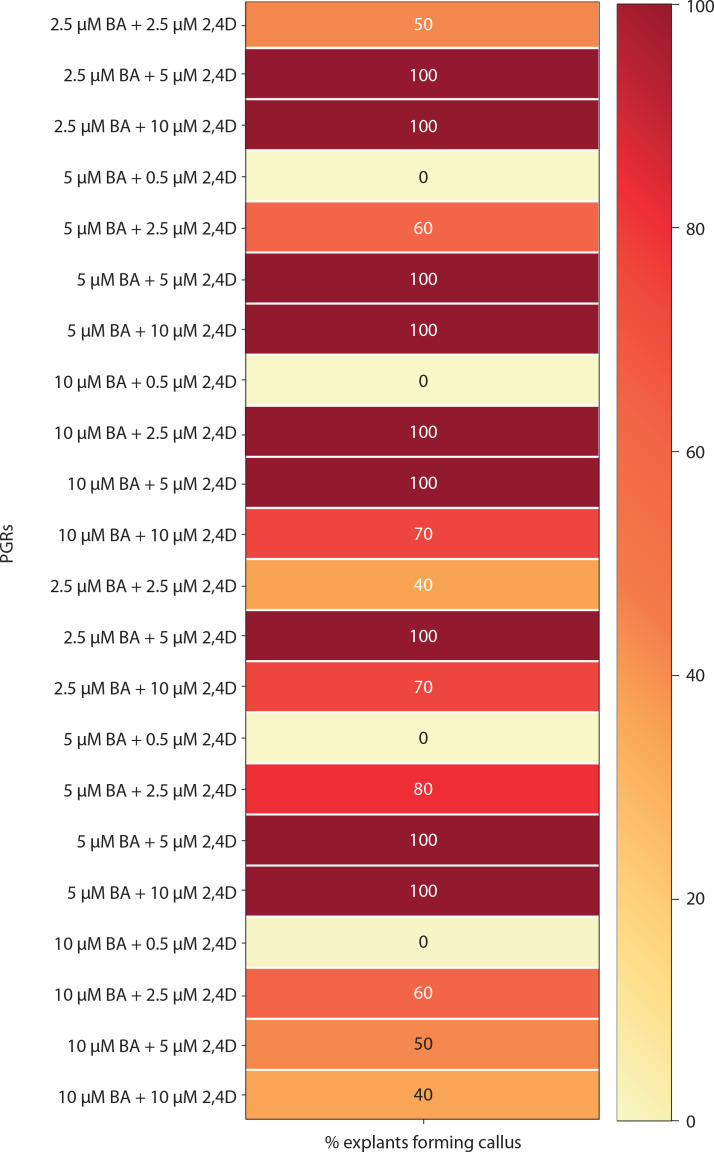
Heat map matrix displaying the combined effect of 6-benzyl adenine (BA)/kinetin (Kin) and 2,4D-supplemented (2,4D) MS medium on the % share of explants forming callus PGRs – plant growth regulators

Medium with BA and NAA induced white-green compact callus and one or two large shoot buds at low concentrations [0.5 μM BA + NAA (2.5–10 μM)] (Figures 4C and 6). At higher concentrations [2.5–10 μM BA + NAA (2.5–10 μM)], only green callus was induced. Medium with Kin and NAA produced little to moderate white-red friable callus (Figures 4D and 6). Combinations of BA (2.5–10 μM) with 0.5 μM Kin did not induce callus, but other combinations formed callus in 40–100% of explants. Moderate to profuse callus was observed on medium supplemented with 5–10 μM BA and 0.5–10 μM 2,4D (Figures 4e and 7). Kin and 2,4D also induced white friable callus in the 1^st^ week of inoculation, which later turned greenish-white (Figures 4F and 7).

Thus, only MS medium supplemented with 10 μM BA and 5 μM IAA supported successful regeneration, where juvenile explants produced shoot buds that proliferated into clusters of small shoots and 2.7 ± 0.15 welldeveloped shoots. All other PGR combinations tested resulted only in callus or shoot buds, none of which developed into mature shoots.

### Anatomy of the regenerating shoots

**Figure 8:**
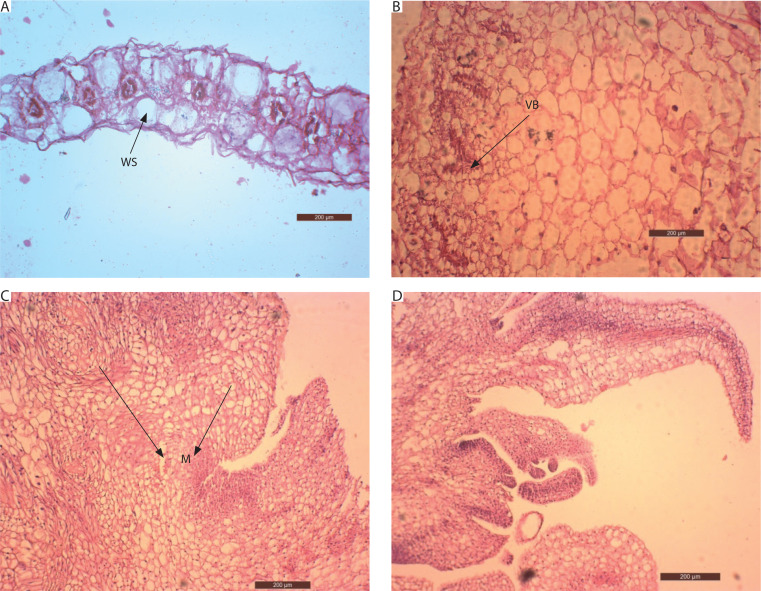
Anatomy of the stages in the development of *in vitro* shoots of *Portulaca grandiflora*. **A**) Transverse section of juvenile leaf on day 0 (200 μm; WS – water storage cells). **B**) Vascular bundles (VB) are pushed towards the periphery, and water storage cells are replaced by parenchymatous cells (200 μm) after 1 week. **C**) Vigorous mitotic division and the cells organize as clusters of meristemoids (M) after 5 weeks (200 μm). The cells are deeply stained with a conspicuous nucleus. **D)** Asynchronous development of apical meristems leading to the formation of *in vitro* shoots after 6 weeks (200 μm)

At the time of inoculation (day 0), the transverse section of *in vivo* juvenile leaves (first nodal leaf of the stem) displayed Kranz anatomy, with a distinct layer of bundle sheath cells surrounding the vascular bundles. Mesophyll cells were clustered around the bundle sheath, and large water-storage cells were present between the upper and lower epidermis (Figure 8A).

Anatomical changes in explants inoculated on MS medium supplemented with 10 μM BA and 5 μM IAA revealed a high rate of cell division. After 7 days, the vascular bundles became distorted and were pushed toward the periphery of the explant. Large water-storage cells were replaced by parenchymatous cells, which occupied the central mass (Figure 8B). By the 4^th^ week, rapid cell division of these parenchymatous cells was evident. The cells were isodiametric, with deeply stained cytoplasm and conspicuous nuclei. They were arranged in clusters, leading to the formation of localized meristemoid regions after 5 weeks (Figure 8C).

Subsequently, these meristemoids were organized into layered structures forming the apical meristem. The apical dome initiated the development of leaf primordia, and shoots emerged from the leaf explants after 6 weeks. Meristems at different developmental stages were observed (Figure 8D). Thus, abundant parenchymatous cell proliferation gave rise to organogenic callus, which initiated shoots through indirect organogenesis.

### Estimation of betalain pigment content

**Figure 9:**
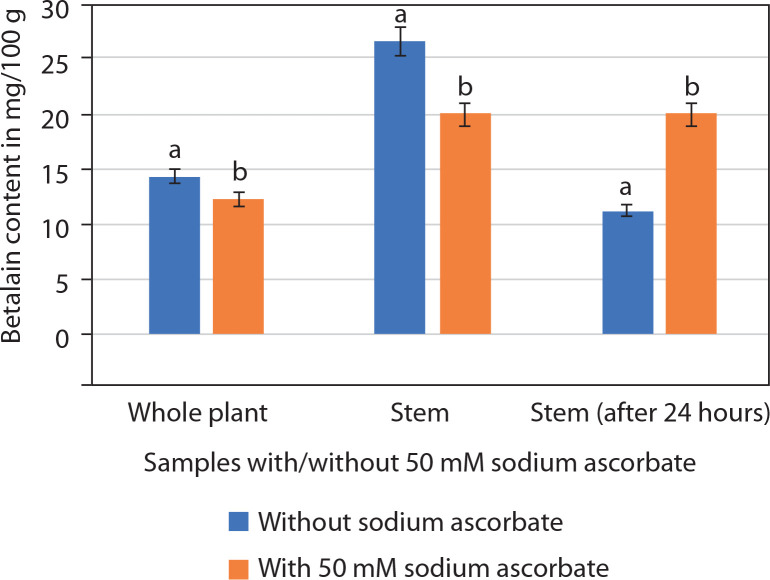
Betalain content (mg/100 g; mean ± SE) in the whole plant and the stem alone of *Portulaca grandiflora* without or with 50 mM sodium ascorbate. Student’s *t*-test was significant at *p* < 0.01 between the samples (without or with 50 mM sodium ascorbate)

Betalains consist of two components: betacyanins and betaxanthins. Spectrophotometric analysis revealed that the betalain extract of *P. grandiflora* (stem/whole plant) contained betacyanins, whereas betaxanthins were absent. Betalain content was higher in the stem (26.66 ± 0.19 mg/100 g) than in the whole plant (Figure 9). Betalain is an unstable pigment that degrades rapidly. Initial analysis showed that both whole plant and stem samples lost pigment within 12 h of storage at 4°C. The addition of 50 mM sodium ascorbate slowed this degradation. In samples without sodium ascorbate, betalain content was 14.35 ± 0.16 mg/100 g (whole plant) and 26.66 ± 0.19 mg/100 g (stem). In contrast, samples treated with 50 mM sodium ascorbate showed 12.35 ± 0.14 mg/100 g (whole plant) and 20.08 ± 0.13 mg/100 g (stem). Thus, betalain content appeared higher in untreated samples than in those with sodium ascorbate (Figure 9). The betalain content of stem samples (with or without sodium ascorbate) was nearly twice that of whole plant samples. Therefore, subsequent analyses focused only on stem samples after 24 h of extraction. These results showed 42.19% degradation in betalain content without sodium ascorbate (11.25 ± 0.62 mg/100 g) compared with samples containing sodium ascorbate (20.11 ± 0.04 mg/100 g; Supplementary Figure 1).

Thus, it is concluded that the stems of *P. grandiflora* are rich in betalains and that sodium ascorbate helps prevent pigment degradation. However, the *in vitro* shoots and calluses did not synthesize sufficient amounts of pigment for quantification.

## Discussion

Regeneration protocols have been developed for several species of *Portulaca* and successfully applied in various biotechnological interventions, such as *Agrobacterium*-mediated transformation and upregulation of different metabolites (Sedaghati et al. 2019; Chen et al. 2020; Srivastava and Joshi 2021). The regeneration protocol developed for *P. grandiflora* in the present study can similarly be used for harvesting important secondary metabolites, such as flavonoids (quercetin) and phenolics (Nurcholis et al. 2023).

Different explants of *Portulaca* show varied responses to PGRs, as demonstrated in several studies. Srivastava and Joshi (2009) and Cruz et al. (2019) reported regeneration from nodal and hypocotyl explants, respectively, using cytokinins (BA/Kin) in *P. grandiflora*. Bhuiyan and Adachi (2002) emphasized that high concentrations of cytokinin (thidiazuron) were required for organogenesis in hypocotyl-derived calli. However, in the present study, cytokinins (BA + Kin) were not effective, as they induced only negligible callus or shoot buds from mature leaf explants, and these shoot buds failed to develop into *in vitro* shoots in cytokinin-rich media.

Previous reports also indicate that regeneration in *P. grandiflora* is favored by a combined concentration of cytokinin and auxin. Safdari and Kazemitabar (2010) reported indirect organogenesis from leaf explants in BA + NAA, and Rossi-Hassani and Zryd (1995) obtained shoots from hypocotyls in the same combination. In contrast, in the present study, explants cultured on BA + NAA produced callus and shoot buds, but these shoot buds failed to proliferate into shoots. This is contrary to the reports of Safdari and Kazemitabar (2010) and Rossi-Hassani and Zryd (1995) in *P. grandiflora*. The combination of BA and IAA induced the development of shoot buds into shoots, indicating that the use of IAA instead of NAA in the medium led to the regeneration of *P. grandiflora* leaf explants (magenta flower phenotype).

The addition of auxins and cytokinins to the medium also influenced the formation and morphology of callus. BA + NAA induced white–green compact callus, Kin + NAA induced white-red callus, while BA/Kin combined with 2,4D induced white friable callus.

A similar observation was reported for *P. grandiflora* by Rossi-Hassani and Zryd (1995), where the color and texture of the callus were determined by the type of PGR used in the medium. Trezzine and Zryd (1990) also reported the formation of a mosaic callus in *P. grandiflora* – a white callus interspersed with red sectors. The present study confirmed the formation of such a heterogeneous mass of tissues (white mass with patches of red tissue) in callus cultures obtained on MS medium supplemented with 5 μM Kin and 2.5 μM NAA.

Similarly, the formation of white-green compact callus from leaf explants in MS medium supplemented with BA and NAA in this study aligns with the findings of Xu et al. (2024) in *P. oleracea*, where leaf explants produced dense, chlorophyll-synthesizing, smooth-textured callus on BA + NAA medium. The interplay of BA/Kin with 2,4D is also known to induce callus in several plants. For example, Srivastava and Joshi (2024) reported a wide spectrum of morphogenic responses in *P. oleracea* when leaf explants were inoculated on MS medium with BA and 2,4D. Their study demonstrated that explants produced 2–3 roots on 0.5 μM BA + 0.5 μM 2,4D; shoot buds and callus on 2.5–5 μM BA + 0.5 μM 2,4D; and well-developed shoots with green callus on 10 μM BA + 0.5 μM 2,4D. A similar pattern of callus and *in vitro* shoot induction was observed in leaf explants supplemented with Kin and 2,4D in the same study (Srivastava and Joshi 2024).

Mousa et al. (2022) also reported callus induction from stem, leaf, and seed explants of *P. oleracea* on MS medium supplemented with BA and 2,4D, where leaf explants produced nodular and compact callus. These reports, however, contrast with the present findings in *P. grandiflora*, where callus generated on MS medium supplemented with BA/Kin and 2,4D was friable and soft and did not undergo organogenesis to produce roots or shoots. Clearly, the response of *Portulaca* to different concentrations and types of PGRs varies among species.

The technique of plant tissue culture is based on totipotency, which implies that any living part (explant) of a plant is capable of dedifferentiation (Long et al. 2022). However, the age of the explant is a crucial factor, as young explants generally exhibit better physiological responses (Kalyan and Sil 2015). In the present study, the performance of juvenile leaf explants was tested on the same medium composition that induced shoots from mature leaf explants. On MS medium supplemented with 10 μM BA and 5 μM IAA, mature leaf explants produced only 2.31 ± 0.86 shoots, whereas juvenile leaf explants consistently yielded 2.7 ± 0.15 well-developed shoots, along with clusters of incipient shoots and shoot buds. Thus, juvenile explants demonstrated a better regenerative response than mature explants.

These findings align with earlier studies. Becerra et al. (2004) reported higher induction of adventitious shoots from juvenile compared with mature leaf explants in *P. edulis*. Long et al. (2022) observed that, regardless of explant type, cell division initiated near the cambium and vascular bundles of young plant parts. Kalyan and Sil (2015) further noted that juvenile tissues possess less rigid cell walls and higher meristematic activity, which promote faster growth. These reports support the present finding that juvenile leaf explants respond more effectively than mature leaf explants.

In the current study, shoot buds developed into shoots on MS medium supplemented with 10 μM BA and 5 μM IAA; however, these buds failed to multiply even under prolonged culture. Therefore, cultures were transferred to the multiplication medium. Cytokinins are known to promote mitotic cell divisions and differentiation of adventitious shoots from callus and organs (Schaller et al. 2014). In the present study, the multiplication of shoots was obtained in the medium supplemented with BA. This is consistent with the findings of Geng et al. (2016), where the addition of cytokinin to the medium induced a faster rate of multiplication of *in vitro* shoots in G30 cultivars of apple. Similarly, Xu et al. (2024) report that the leaf explant of *P. oleracea* induced callus in MS medium supplemented with BA and NAA. This callus, which, when transferred to MS medium supplemented with BA, resulted in the development of multiple shoots.

Thus, BA supplementation has a high potential to induce rapid cell division, leading to the development and multiplication of *in vitro* shoots. The present study confirms that MS medium supplemented with BA is effective for *in vitro* shoot multiplication in *P. grandiflora*.

GA_3_ is commonly used for *in vitro* shoot elongation. Based on the findings of Geng et al. (2016) in apple, it was concluded that GA_3_ in combination with BA promoted both elongation and multiplication of *in vitro* shoots, which contrasts with our results. Little and Macdonald (2003) reported that GA_3_ stimulates the subapical meristem in newly developed shoots of *Pinus*; as such, GA_3_ does not increase shoot numbers but promotes elongation. Our findings are consistent with this phenomenon, as the number of shoots did not increase appreciably, but elongation was observed in basal MS medium supplemented with GA_3_.

The anatomy of the regenerating shoots was examined to establish the mode of regeneration (direct/indirect organogenesis) in this plant via leaf explants. The anatomical studies have established a series of stages that lead to the development of *in vitro* shoots in culture systems.

The transverse section of *P. grandiflora* leaves at day 0 showed vascular bundles surrounded by large bundle sheath cells, a feature known as Kranz anatomy, characteristic of C_4_ plants (Guralnick et al. 2020). These vascular bundles, arranged laterally, were encircled by large water-storage cells. A similar anatomical pattern was described in *P. grandiflora* leaves by Voznesenskaya et al. (2010) and Guralnick et al. (2020), who referred to it as the “pilosoid type of Kranz anatomy.”

Shoot organogenesis involves the formation of clusters of rapidly dividing, thin-walled meristematic cells, characterized by dense cytoplasmic staining and prominent nuclei. These clusters have been variously termed meristemoids, pro-meristems, or meristemoidlike precursors and are a common feature of shoot organogenesis (Schuchovski et al. 2020). The present study demonstrated indirect organogenesis, where shoots originated from callus cells, consistent with the reports of Schuchovski et al. (2020). Asynchronous development of leaf primordia was also observed on the apical meristem, corroborating earlier findings by Schuchovski et al. (2020) and Woo and Wetzstein (2008).

Thus, the present study illustrates the sequential anatomical stages underlying *in vitro* shoot development in *P. grandiflora* culture systems.

*P. grandiflora* is rich in betalains, which impart a deep red color to its stem. Betalains are derived from a core betalamic acid, which forms betacyanins through imino linkage with cyclo-DOPA or betaxanthins through conjugation with an amine (Polturak and Aharoni 2018). These pigments are restricted to the order Caryophyllales in plants and to certain basidiomycetes in fungi (Timoneda et al. 2019). Owing to their bright coloration, betalains attract insects for pollination and seed dispersal. In addition, they function as secondary metabolites involved in plant defense mechanisms (Polturak and Aharoni 2018).

Trezzini and Zryd (1991) reported the presence of betaxanthins (Portulacaxanthin III) in the petals of red, yellow, and orange phenotypes of *P. grandiflora*, but not in the white flower phenotype. Gandia-Harrero et al. (2005) detected betanidin (a precursor of betacyanin) in the violet variety and betaxanthins in the yellow variety, whereas in the white-flowered phenotype, only negligible amounts of betanidin were present and betaxanthins were absent. Similarly, Trezzini and Zryd (1990) found that plants with violet-colored flowers and stems contained betacyanins in the stem but no betaxanthins. More recently, Spórna-Kucab et al. (2022) reported that yellow and orange flowers of *P. grandiflora* had the highest betaxanthin content but significantly lower levels of betacyanins, while purple flowers were particularly rich in betacyanins.

The present study reported the presence of betacyanins and the absence of betaxanthins in the stems of *P. grandiflora* with a deep magenta flower phenotype. Flower color is, therefore, an indicator of the type of pigment that can be isolated from the plant: yellow or orange flowers yield betaxanthins, whereas pink or magenta flowers are rich in betacyanins. It was also observed that betalain content was higher in stem samples compared to whole-plant samples. The whole plant consisted of root, leaves, and stems, while the stemcontained only stem tissue, excluding the nonpigmented roots and leaves. Both samples had the same biomass weight (10 g). The leaves and roots did not have betalain pigment. Thus, in the whole plant, the biomass contributed by leaves and roots did not contribute to pigment quantity. Understandably, the pigment content is higher in only the stem sample rather than the whole plant.

Betalains are highly unstable and sensitive to changes in pH, temperature, and light exposure. Pigment stability can be enhanced by the addition of ascorbic acid, which lowers pH and prevents oxidation by inhibiting polyphenol oxidase activity (Guerrero-Rubio et al. 2019). Studies on *Chenopodium quinoa* (Escribano et al. 2017) and *Parakeelya mirabilis* (Chung et al. 2015) reported that 50 mM sodium ascorbate enhanced betalain stability. The present findings are in agreement with these reports, as the addition of 50 mM sodium ascorbate to samples prevented betalain degradation after 24 h.

Further, Vieira Teixeira da Silva et al. (2019) demonstrated that betalains in highly purified beetroot extracts remained stable for 9 months at –30°C and for 20 days at 4°C. These findings highlight the importance of strict control of temperature, pH, and light exposure during purification, which significantly reduces pigment degradation. Thus, achieving betalain stability requires a holistic approach in which the activity of sodium ascorbate is enhanced by strict control over temperature, pH, and light exposure.

In contrast, appreciable amounts of betalains were not synthesized within *in vitro* cultures in the present study. Georgiev et al. (2008) noted that betalain yield in *in vitro* cultures is generally low and is influenced by multiple physical and chemical factors, including cultivar type, subculture duration, and nutrient medium composition. Cytokinins have also been reported to exert an antagonistic effect on betalain production (Santos-Díaz et al. 2005).

Comparative studies show wide variation in betalain content across plants. Silva et al. (2020) reported 75 mg/g betalains in red beet, and Swamy et al. (2014) reported 30.9 mg/100 g in beetroot. In the present study, the stem of *P. grandiflora* contained 26.66 ± 0.19 mg/100 g betalains. Moreover, the presence of amino acids such as tyrosine and leucine, as well as elicitors like silver nitrate, has been shown to enhance betalain content (Winson et al. 2021).

## Conclusions

In the present study, a regeneration protocol from leaf explants was developed for *P. grandiflora*. This three-step procedure, involving shoot bud induction, shoot multiplication, and shoot elongation, proved to be reliable and reproducible. Anatomical studies confirmed that regeneration of *in vitro* shoots followed an indirect pathway. A high content of betalains was detected in the stems of garden-grown plants, and rapid degradation of the pigment was prevented by the addition of sodium ascorbate. In contrast, *in vitro* shoots and callus cultures did not accumulate appreciable amounts of betalains. Future investigations could focus on enhancing betalain accumulation within *in vitro* plants or callus cultures, for which the developed regeneration protocol provides a useful platform. Additionally, the influence of abiotic factors such as temperature, pH, and light on betalain synthesis should be explored to further improve pigment stability.

## References

[cit0001] Anghel AI, Olaru OT, Gatea F, Dinu M, Ancuceanu RV, Istudor V. 2013. Preliminary research on Portulaca grandiflora Hook. species (Portulacaceae) for therapeutic use. Farmacia. 61(4): 694–702.

[cit0002] Azni MM, Moradi H, Ghasemi K, Biparva P. 2021. Elicitation of dopamine biosynthesis in common purslane as affected by methyl jasmonate and silicon. J Plant Nutr. 44(20): 3083–3098. 10.1080/01904167.2021.1936027.

[cit0003] Becerra DC, Forero AP, Gongora GA. 2004. Age and physiological condition of donor plants affect in vitro morphogenesis in leaf explants of Passiflora edulis f. flavicarpa. Plant Cell Tiss Organ Cult. 79: 87–90. 10.1023/B:TICU.0000049440.10767.29.

[cit0004] Bhuiyan MNH, Adachi T. 2002. Efficient regeneration from hypocotyl cultures of betalain-forming plant Portulaca sp. cv. ‘Jewel’. Stimulatory effect of thidiazuron. Plant Biotechnol. 19(1): 57–61.

[cit0005] Calvi P, Terzo S, Amato A. 2022. Betalains: colours for human health. Nat Prod Res. 1–20. 10.1080/14786419.2022.2106481. 35921318

[cit0006] Chen S, Xiong Y, Yu X, Pang J, Zhang T, Wu K, Ren H, Jian S, Teixeira da Silva JA, Xiong Y, et al. 2020. Adventitious shoot organogenesis from leaf explants of Portulaca pilosa L. Sci Rep. 10: 3675. 10.1038/s41598-020-60651-w. 32111887 PMC7048842

[cit0007] Chung HH, Schwinn KE, Ngo HM, Lewis DH, Massey B, Calcott KE, Crowhurst R, Joyce DC, Gould KS, Davies KM, Harrison DK. 2015. Characterization of betalain biosynthesis in Parakeelya flowers identifies the key biosynthetic gene DOD as belonging to an expanded LigB gene family that is conserved in betalain-producing species. Front Plant Sci. 6: 499. 10.3389/fpls.2015.00499. 26217353 PMC4493658

[cit0008] Cruz CF, Santos WF, Souza CS, Machado MD, Carvalho IF, Rocha DI, Silva ML. 2019. In vitro regeneration and flowering of Portulaca grandiflora Hook. Ornamental Hortic. 25(4): 443–449. 10.1590/2447-536X.v25i4.2077.

[cit0009] El-Ashry AAE, El-Bahr MK, Gabr AMM. 2020. Effect of light quality on betalain content of red beet (Beta vulgaris L.) cultured in vitro. Egypt Pharm J. 19(2): 143–148. 10.4103/epj.epj_43_19.

[cit0010] Escribano J, Cabanes J, Jiménez-Atiénzara M, Ibańez-Tremolada M, Gómez-Pando LR, García-Carmona F, Gandía-Herrero F. 2017. Characterization of betalains, saponins and antioxidant power in differently colored quinoa (Chenopodium quinoa) varieties. Food Chem. 234: 285–291. 10.1016/j.foodchem.2017.04.187. 28551238

[cit0011] Gandía-Herrero F, Escribano J, García-Carmona F. 2005. Betaxanthins as pigments responsible for visible fluorescence in flowers. Planta. 222(4): 586–593. 10.1007/s00425-005-0004-3. 16177911

[cit0012] Geng F, Moran R, Day M, Halteman W, Zhang D. 2016. Increasing in vitro shoot elongation and proliferation of ‘G.30’ and ‘G.41’ apple by chilling explants and plant growth regulators. HortScience. 51(7): 899–904. 10.21273/HORTSCI.51.7.899.

[cit0013] Georgiev V, Ilieva M, Bley T, Pavlov A. 2008. Betalain production in plant in vitro systems. Acta Physiol Plant. 30: 581–593. 10.1007/s11738-008-0170-6.

[cit0014] Gilbert MG, Phillips SM. 2000. A review of the opposite leaved species of Portulaca in Africa and Arabia. Kew Bull. 55: 769–802. 10.2307/4113627.

[cit0015] Guerrero-Rubio MA, Hernández-García S, García-Carmona F, Gandía-Herrero F. 2019. Extension of life-span using a RNAi model and in vivo antioxidant effect of Opuntia fruit extracts and pure betalains in Caenorhabditis elegans. Food Chem. 274: 840–847. 10.1016/j.foodchem.2018.09.067. 30373018

[cit0016] Guralnick LJ, Gilbert K, Denio D, Antico N. 2020. The development of Crassulacean acid metabolism (CAM) photosynthesis in cotyledons of the C4 species, Portulaca grandiflora (Portulacaceae). Plants. 9(1): 55. 10.3390/plants9010055. 31906418 PMC7020464

[cit0017] Johansen DA. 1940. A plant microtechnique. London: McGraw-Hill Book Company, Inc.

[cit0018] Kalyan K, Sil S. 2015. Proto corm-like bodies and plant regeneration from foliar explants of Coelogyne flaccida, a horticulturally and medicinally important endangered orchid of Eastern Himalaya. Lankesteriana. 15: 151–158. 10.15517/lank.v15i2.20747.

[cit0019] Kugler F, Stintzing FC, Carle R. 2004. Identification of betalains from petioles of differently colored Swiss chard (Beta vulgaris L. ssp. cicla [L.] Alef. cv. Bright Lights) by high-performance liquid chromatography-electrospray ionization mass spectrometry. J Agric Food Chem. 52(10): 2975–2981. 10.1021/jf03591w. 15137842

[cit0020] Little CHA, Macdonald JE. 2003. Effects of exogenous gibberellin and auxin on shoot elongation and vegetative bud development in seedlings of Pinus sylvestris and Picea glauca. Tree Physiol. 23: 73–83. 10.1093/treephys/23.2.73. 12533302

[cit0021] Long Y, Yang Y, Pan G, Shen Y. 2022. New insights into tissue culture plant-regeneration mechanisms. Front Plant Sci. 13: 926752. 10.3389/fpls.2022.926752. 35845646 PMC9280033

[cit0022] McIlvaine T. 1921. A buffer solution for colorimetric comparison. J Biol Chem. 49: 183–186. 10.1016/S0021-9258(18)86000-8.

[cit0023] Moghadam YA, Piri Kh, Bahramnejad B, Ghiasvand T. 2014. Dopamine production in hairy root cultures of Portulaca oleracea (purslane) using Agrobacterium rhizogenes. J Agric Sci Technol. 16: 409–420.

[cit0024] Moreno Sanz P, D’Amato D, Nebish A, Costantini L, Grando MS. 2020. An optimized histological procedure to study the female gametophyte development in grapevine. Plant Methods. 16: 61. 10.1186/s13007-020-00604-6. 32377221 PMC7195713

[cit0025] Mousa AM, Abd-ElShafy E, Bedair R, Khafagi OMA, Abou Elkhier ZA. 2022. Callus induction and influence of biotic and abiotic elicitation on active constituents of Portulaca oleracea L. calli induced in vitro. Egypt J Chem. 65(11): 29–40. 10.21608/ejchem.2022.132440.5846.

[cit0026] Murashige T, Skoog F. 1962. A revised medium for rapid growth and bio assays with tobacco tissue cultures. Physiol Plant. 15: 473–497.

[cit0027] Nurcholis W, Aisyah SI, Saraswati ARM, Yudha YS. 2023. Total phenolics, flavonoid contents, and antioxidant activity of three selected Portulaca grandiflora mutants in MV8 generations as a result of recurrent irradiation technique. J Appl Biol Biotechnol. 11(3): 245–249. 10.7324/JABB.2023.111110.

[cit0028] Polturak G, Aharoni A. 2018. “La Vie en Rose”: Biosynthesis, source, and applications of betalain pigments. Mol Plant. 11(1): 7–22. 10.1016/j.molp.2017.10.008. 29081360

[cit0029] Rossi-Hassani BD, Zryd JP. 1995. In vitro culture and plant regeneration of large flowered purslane. Plant Cell Tiss Organ Cult. 41: 281–283. 10.1007/BF-00045093.

[cit0030] Safdari Y, Kazemitabar SK. 2009. Plant tissue culture study on two different races of purslane (Portulaca oleracea L.). Afr J Biotechnol. 8(21): 5906–5912.

[cit0031] Safdari Y, Kazemitabar SK. 2010. Direct shoot regeneration, callus induction and plant regeneration from callus tissue in moss rose (Portulaca grandiflora L.). Plant Omics J. 3(2): 45–51.

[cit0032] Santos-Diaz MS, Velásquez-García Y, González-Chávez MM. 2005. Pigment production by callus of Mammillaria candida Scheidweiler (Cactaceae). Agrociencia. 39: 619–626.

[cit0033] Schaller GC, Street IH, Kieber JJ. 2014. Cytokinin and the cell cycle. Curr Opin Plant Biol. 21: 7–15. 10.1016/j.pbi.2014.05.015. 24994531

[cit0034] Schuchovski C, Sant’Anna-Santos BF, Marra RC, Biasi LA. 2020. Morphological and anatomical insights into de novo shoot organogenesis of in vitro ‘Delite’ rabbiteye blueberries. Heliyon. 6(11): e05468. 10.1016/j.heliyon.2020.e05468. 33251355 PMC7677692

[cit0035] Sedaghati B, Haddad R, Bandehpour M. 2019. Efficient plant regeneration and Agrobacterium-mediated transformation via somatic embryogenesis in purslane (Portulaca oleracea L.), an important medicinal plant. Plant Cell Tiss Organ Cult. 136: 231–245. 10.1007/s11240-018-1509-3.

[cit0036] Silva JPP, Bolanho BC, Stevanato N, Massa TB, da Silva C. 2020. Ultrasound-assisted extraction of red beet pigments (Beta vulgaris L.): Influence of operational parameters and kinetic modelling. J Food Process Preserv. 44: e14762. 10.1111/jfpp.14762.

[cit0037] Spórna-Kucab A, Tekieli A, Grzegorczyk A, Świątek Ł, Rajtar B, Skalicka-Woźniak K, Starzak K, Nemzer B, Pietrzkowski Z, Wybraniec S. 2022. Metabolite profiling analysis and the correlation with biological activity of betalain-rich Portulaca grandiflora Hook. extracts. Antioxidants. 11: 1654. 10.3390/antiox11091654. 36139728 PMC9495615

[cit0038] Srivastava A, Joshi A. 2009. In vitro behavior of nodal explants of Portulaca grandiflora under the influence of cytokinins. Acta Univ Latviensis. 753: 43–48.

[cit0039] Srivastava A, Joshi A. 2021. Fatty acid analysis of in vitro shoot cultures of Portulaca oleracea Linn. Plant Physiol Rep. 26: 321–328. 10.1007/s40502-021-00582-4.

[cit0040] Srivastava A, Joshi A. 2024. Optimization of callus induction protocol from leaf explants of Portulaca oleracea and assessment of fatty acid profiles. Environ Exp Biol. 22: 157–166. 10.22364/eeb.22.15.

[cit0041] Swamy GJ, Sangamithra A, Chandrasekar V. 2014. Response surface modeling and process optimization of aqueous extraction of natural pigments from Beta vulgaris using Box–Behnken design of experiments. Dyes Pigments. 111: 64–74. 10.1016/j.dyepig.2014.05.028.

[cit0042] Timoneda A, Feng T, Sheehan H, Walker-Hale N, Pucker B, Lopez-Nieves S, Guo R, Brockington S. 2019. The evolution of betalain synthesis in Caryophyllales. New Phytol. 224: 71–85. 10.1111/nph.15980. 31172524

[cit0043] Trezzine GF, Zryd JP. 1991. Two betalains from Portulaca grandiflora. Phytochemistry. 30(6): 1897–1899. 10.1016/0031-9422(91)85035-X.

[cit0044] Trezzini GF, Zryd JP. 1990. Portulaca grandiflora: A model system for the study of the biochemistry and genetics of betalain synthesis. Acta Hortic. 280: 581–585. 10.17660/ActaHortic.1990.280.95.

[cit0045] Vieira Teixeira da Silva D, Dos Santos Baião D, de Oliveira Silva F, Alves G, Perrone D, Mere Del Aguila E, Flosi Paschoalin VM. 2019. Betanin, a natural food additive: Stability, bioavailability, antioxidant and preservative ability assessments. Molecules. 24: 458. 10.3390/molecules24030458. 30696032 PMC6384587

[cit0046] Voznesenskaya EV, Koteyeva NK, Edwards GE, Ocampo G. 2010. Revealing diversity in structural and biochemical forms of C4 photosynthesis and a C3–C4 intermediate in genus Portulaca L. (Portulacaceae). J Exp Bot. 61(13): 3647–3662. 10.1093/jxb/erq178. 20591900 PMC2921202

[cit0047] Wang C, Yu J, Gallagher DL, Byrd J, Yoo W, Wang Q, Guo O, Dietrich AM, Yang M. 2020. Pyrazines: A diverse class of earthy-musty odorants impacting drinking water quality and consumer satisfaction. Water Res. 182: 115971. 10.1016/j.watres.2020.115971. 32554269

[cit0048] Wang T, Liu L, Rakhmanova A, Wang X, Shan Y, Yi Y, Liu B, Zhou Y, Lu X. 2020. Stability of bioactive compounds and in vitro gastrointestinal digestion of red beetroot jam: effect of processing and storage. Food Biosci. 38: 100788. 10.1016/j.fbio.2020.100788.

[cit0049] Winson KWS, Chew BL, Sathasivam K, Subramaniam S. 2021. Effect of amino acid supplementation, elicitation and LEDs on Hylocereus costaricensis callus cultures for the enhancement of betalain pigments. Sci Hortic. 289: 110459. 10.1016/j.scienta.2021.110459.

[cit0050] Woo S, Wetzstein HY. 2008. Morphological and histological evaluation of in vitro regeneration in Elliottia racemosa leaf explants induced on media with thidiazuron. J Am Soc Hortic Sci. 133(2): 167–172. 10.21273/JASHS.133.2.167.

[cit0051] Xu M, Zhao X, Fang J, Yang Q, Li J, Yan J. 2024. An effective somatic-cell regeneration and genetic transformation method mediated by Agrobacterium tumefaciens for Portulaca oleracea L. Plants. 13: 2390. 10.3390/plants13172390. 39273876 PMC11396874

